# Divergent RUNX2-Associated Regulation of FAM20A and FAM83H in the Enamel–Eruption Developmental Axis

**DOI:** 10.3390/ijms27209222

**Published:** 2026-10-17

**Authors:** Songya Mao, Siqi Wei, Hui Jia, Yuzhuan Hou, Wenyan Ruan, Jing Dong, Jialin Xu, Xiaohong Duan

**Affiliations:** 1 State Key Laboratory of Oral & Maxillofacial Reconstruction and Regeneration, National Clinical Research Center for Oral Disease, Shaanxi Key Laboratory of Stomatology, Department of Oral Biology, Clinic of Oral Rare Diseases and Genetic Diseases, School of Stomatology, The Fourth Military Medical University, Xi’an 710032, China; 2 Department of Orthodontic, School of Stomatology, The Fourth Military Medical University, Xi’an 710032, China

**Keywords:** amelogenesis imperfecta, tooth eruption, FAM20A, FAM83H, RUNX, dental follicle

## Abstract

Amelogenesis imperfecta (AI) associated with family with sequence similarity 20 member A (FAM20A) and family with sequence similarity 83 member H (FAM83H) may be accompanied by both enamel defects and tooth-eruption abnormalities; however, the tissue-level and molecular mechanisms underlying the relationship between these phenotypes remain poorly understood. To explore the mechanism, here we studied seven AI individuals from three families by oral and radiographic examination and whole-exome or whole-genome sequencing. Eruption abnormalities in FAM20A- and FAM83H-related AI were analyzed by integrating these seven cases with our previous retrospective datasets and newly reported cases from the literature. FAM20A and FAM83H expression was examined by RNA sequencing of human dental tissues and by immunohistochemistry in human dental follicle tissue and P0/P7 mouse tooth germs. RUNX2 regulation of *FAM20A* and *FAM83H* was assessed by CUT&Tag in dental follicle and *RUNX2* siRNA knockdown. Genetic analysis identified two distinct heterozygous pathogenic truncating *FAM83H* variants in six patients from two families and compound heterozygous *FAM20A* variants in one patient from the third family. Eruption abnormalities were observed in the *FAM20A* patient and four of the six patients with *FAM83H* variants. In the combined dataset, eruption abnormalities were documented in 36 of 37 (97.3%) evaluable FAM20A cases and 18 of 58 (31.0%) evaluable FAM83H cases. In human dental follicle tissues surrounding impacted third molars, both proteins localized predominantly to epithelial lining components. In developing mouse tooth germs, both proteins were enriched in odontogenic epithelial/enamel-organ compartments with weaker staining in mesenchymal compartments. Dental-follicle RNA profiling further revealed an epithelial/keratin signature and detectable *FAM20A*/*FAM83H* expression. CUT&Tag identified RUNX2 binding at *FAM20A* but not *FAM83H*, and *RUNX2* knockdown reduced *FAM20A* expression without a corresponding decrease in *FAM83H*. We conclude that *FAM20A*- and *FAM83H*-associated AI exhibit distinct patterns of eruption abnormalities in addition to enamel defects. Our findings support *FAM20A* as a candidate RUNX2-regulated target in dental follicle cells, whereas no comparable regulatory relationship was evident for *FAM83H* under the experimental conditions.

## 1. Introduction

Amelogenesis imperfecta (AI) comprises a genetically heterogeneous group of inherited disorders characterized primarily by quantitative or qualitative defects in enamel formation [1,2,3]. Some forms of AI also exhibit abnormalities beyond enamel, particularly delayed or failed tooth eruption [4,5,6]. Furthermore, we found that these tooth-eruption failures exhibit gene-dependent patterns and termed this phenotype selective failure of tooth eruption (SFTE) [4,5]. Among AI-associated genes, *FAM20A* (formerly family with sequence similarity 20 member A; FAM20A Golgi associated secretory pathway pseudokinase) and *FAM83H* (formerly family with sequence similarity 83 member H; now SACK1H, scaffolding CK1 anchoring protein H) are particularly relevant to SFTE, as pathogenic variants in both genes disrupt enamel formation and can also impair tooth eruption. FAM20A-associated enamel-renal syndrome (ERS) is characterized by extensive eruption failure [6, 7, 8, 9], whereas FAM83H-associated autosomal dominant hypocalcified AI (ADHCAI) [10] exhibits a more limited eruption phenotype, frequently involving the canines and second molars [4,11]. These findings indicate that two genetically and phenotypically distinct forms of AI can both involve abnormal eruption, suggesting that enamel-associated genes may also participate in the biological processes governing tooth eruption.

Tooth eruption is a tightly regulated developmental process requiring coordinated interactions among odontogenic tissues, the dental follicle, osteoclasts, and osteoblasts [5]. The coexistence of enamel defects and eruption failure raises an important question: whether these phenotypes are independent consequences of genetic defects or reflect a shared developmental mechanism. FAM20A has established roles in enamel biomineralization [8,12] and is expressed in ameloblast-lineage epithelium, whereas FAM83H is associated with epithelial cytoskeletal and junctional organization [13]. Previous studies have implicated FAM83H in osteogenic differentiation, craniofacial bone development, and osteosarcoma progression, with evidence suggesting the involvement of Wnt/β-catenin signaling [14,15,16]. Although the dental follicle is predominantly ectomesenchymal and coordinates osteoclast recruitment, osteogenesis, and eruption-pathway formation [17], the dental follicle may also contain reduced enamel epithelium and persistent odontogenic epithelial remnants [18]. Whether these epithelial components remain biologically active during human tooth eruption and how FAM20A and FAM83H relate to the epithelial and mesenchymal compartments of the dental follicle remain unclear.

RUNX2 represents a potential regulatory link in this context. As a key transcriptional regulator of dental follicle and bone biology, RUNX2 contributes to osteogenic differentiation, osteoclast-inductive signaling, and eruption-pathway formation [19,20], and impaired RUNX2 function is strongly associated with delayed or failed tooth eruption in cleidocranial dysplasia (CCD) [21]. However, whether a regulatory relationship exists between RUNX2 and FAM20A or FAM83H in the dental follicle remains unknown.

In the present study, we integrated clinical, genetics, retrospective genotype–phenotype analysis, dental follicle transcriptomics, histological localization, CUT&Tag profiling, and *RUNX2* knockdown to investigate the relationship between amelogenesis and tooth eruption. Through this integrated approach, we sought to characterize the localization of FAM20A and FAM83H in odontogenic epithelial and follicular compartments and to examine their potential relationships with tooth eruption and RUNX2-associated regulation.

## 2. Results

### 2.1. FAM83H-Associated ADHCAI: Hypocalcified Enamel with Selective Eruption Abnormalities

Six affected individuals from two families showed characteristic AI enamel defects and carried the pathogenic variants in *FAM83H*, and four of them (66.7%) showed at least one tooth-eruption abnormality in addition to the enamel phenotype.

In Family 1, the proband was 26 years old and has undergone full-mouth restoration with porcelain crowns. She has four affected adult relatives showing an autosomal dominant pattern of generalized yellow to yellow-brown enamel discoloration, rough hypomineralized surfaces, post-eruptive enamel loss, and attrition. Panoramic imaging showed reduced enamel radiodensity in the erupted and unerupted teeth in all five cases. Clinically relevant non-third-molar eruption failure included unerupted canines in the proband (III2: 33 and 43) and other two affected relatives (III1: 43; III3: 23) (Figure 1A). In Family 2, a 16-year-old boy had generalized rough yellow-brown enamel, extensive post-eruptive enamel loss, and groove-like defects. His tooth 27 showed eruption failure on panoramic imaging (Figure 1B).

Whole-exome sequencing identified the heterozygous variant in *FAM83H* NM_198488.5: c.2029C>T [p.(Gln677Ter)] in the AI cases of Family 1 (Figure 1A). Sanger sequencing confirmed the variant in all five tested affected individuals, whereas it was absent in the tested unaffected relatives. In Family 2, the proband carried the heterozygous variant in *FAM83H* NM_198488.5: c.1192C>T [p.(Gln398Ter)]; his unaffected mother was variant-negative, while his father exhibited a similar AI phenotype but was unavailable for genetic testing (Figure 1B). Both variants are located in exon 5, the terminal coding exon of *FAM83H*, within the established ADHCAI-associated truncation region spanning approximately Ser287–Glu694. They have both been previously reported in ADHCAI and are classified as pathogenic in ClinVar [10,22,23,24].

### 2.2. FAM20A-Associated ERS: Severe Enamel Hypoplasia, Eruption Failure, and Ectopic Mineralization

The proband was a 33-year-old woman who had undergone full-mouth prosthodontic rehabilitation. Intraoral examination showed no obvious gingival overgrowth. Panoramic and CBCT imaging showed failed eruption of teeth 25, 28, 35, 37, 38, and 48 (Figure 2A). The unerupted teeth (Teeth 35 and 37) had pulp calcifications, root dysplasia and incomplete crown with an irregular, eroded contour (Figure 2A). Based on the characteristic dental phenotype and renal CT findings of multiple punctate hyperdense foci in both renal pelves, the proband was diagnosed with ERS.

Whole genome sequencing identified two *FAM20A* variants based on the reference transcript NM_017565.4: c.755_757delTCT [p.(Phe252del)] and c.826C>T [p.(Arg276Ter)] (Figure 2B), which were confirmed to be in trans by cloning-based allelic separation (Figure 2C,D). The p.(Arg276Ter) nonsense variant is an established pathogenic allele, whereas the in-frame p.(Phe252del) variant has previously been reported in affected individuals and classified as likely pathogenic [7,12]. Functional and structural studies suggest that p.Phe252del affects a region critical for FAM20A dimerization and FAM20A–FAM20C interaction, thereby impairing protein function [25,26].

### 2.3. Retrospective Analysis of FAM20A- and FAM83H-Associated Eruption Abnormalities

To examine the gene-specific eruption patterns in a combined dataset, we combined our previous datasets [4,5] with subsequently reported genetically confirmed cases [27,28,29,30,31,32] and the three families in the present study (Table 1). The frequencies reported below represent descriptive proportions among cases with evaluable eruption data rather than estimates of phenotype penetrance.

For FAM20A, the historical cohort comprised 20 patients, all of whom had reported eruption failure (100%), involving 209 unerupted teeth (mean, 10.5 per patient) [5]. Among the newly reported cases, eruption status could be determined individually in 16 patients, of whom 15 (93.8%) had delayed or failed eruption. After inclusion of the present *FAM20A*-associated patient, eruption abnormalities were identified in 36 of 37 individually evaluable patients (97.3%). Across these cases, eruption abnormalities commonly involved multiple unerupted or impacted teeth, often accompanied by pulp, follicular, or root abnormalities (Table 1).

For FAM83H, the historical dataset included 50 patients, of whom 12 (24.0%) had reported eruption abnormalities involving 34 unerupted teeth, with canines and second molars most frequently affected [4]. After inclusion of two newly published patients and six AI cases from our present families, eruption abnormalities were documented in 18 of 58 evaluable patients (31.0%) (Table 1).

Overall, the combined dataset showed distinct gene-specific eruption patterns, with abnormalities documented in all evaluable *FAM20A*-associated cases and in a smaller proportion of *FAM83H*-associated cases, with differences in the number and distribution of affected teeth.

### 2.4. Exploratory Transcriptomic Profiling of Human Dental Follicle, Dental Pulp, and Apical Papilla

To explore potential tissue-level links between enamel defects and tooth-eruption abnormalities, we compared epithelial-associated transcripts across dental follicle, dental pulp, and apical papilla. The total mRNA was collected from a healthy volunteer undergoing extraction of an impacted third molar and analyzed by bulk RNA sequencing. *FAM20A* and *FAM83H* transcripts were both detectable in the dental-follicle dataset although lower than in the other two tissues. However, comparison of transcript abundance showed that the dental-follicle sample was enriched for keratin and other epithelial-associated transcripts relative to dental pulp and apical papilla. The most highly expressed epithelial-associated genes in the dental follicle were visualized by FPKM comparison and heat-map analysis (Figure 3). These findings provided molecular evidence that *FAM20A*/*FAM83H* and epithelial components located dental follicle tissue.

### 2.5. Tissue Localization of FAM83H and FAM20A in Human Dental Follicles and Developing Mouse Teeth

To further characterize the tissue distribution of FAM20A and FAM83H in eruption-associated tissues, both proteins were examined by immunohistochemistry in human dental follicles and in mouse tooth germs at postnatal day 0 (P0) and postnatal day 7 (P7).

In human dental follicle tissue surrounding an impacted third molar, strong FAM83H immunoreactivity was observed in the odontogenic epithelial remnants, whereas the surrounding follicular connective tissue showed weak staining (Figure 4A). Strong FAM83H immunoreactivity was also observed in the epithelial lining of a dentigerous cyst from the maxillary canine region (Figure 4B), representing a pathological epithelial compartment rather than normal developmental odontogenic epithelium. In mouse tooth germs, FAM83H was detected mainly in oral epithelium, major epithelial components of the enamel organ, ameloblasts, and odontoblasts. Dental-follicle staining was weak at P0 (Figure 4C) and was not prominent at P7 (Figure 4D). Overall, FAM83H showed predominantly epithelial localization in the examined tissues.

FAM20A showed a partially overlapping but broader distribution (Figure 5). In P0 and P7 mouse tooth germs, strong FAM20A immunoreactivity was observed in oral and odontogenic epithelium and in enamel-organ/ameloblast-lineage cells. Weak staining was also present in mesenchymal compartments, including dental papilla, odontoblasts, dental follicle, and adjacent bone (Figure 5C,D). In human dental follicles surrounding an impacted third molar, FAM20A showed strong epithelial and weak connective-tissue staining (Figure 5A). Strong staining was also observed in the pathological epithelial lining of a dentigerous cyst (Figure 5B). Overall, FAM20A showed predominantly epithelial localization, with weaker staining in mesenchymal and mineralizing tissues.

Within the dental follicle, both FAM83H and FAM20A exhibited prominent staining in epithelial regions, whereas relatively weak staining was observed in the surrounding mesenchymal regions.

### 2.6. RUNX2 CUT&Tag Identifies RUNX2 Occupancy at FAM20A Regulatory Regions

Given the essential role of RUNX2 in dental follicle function and tooth eruption, we investigated whether FAM20A or FAM83H represents a candidate RUNX2-regulated target of RUNX2 in the dental follicle. RUNX2 CUT&Tag profiling was therefore performed using dental follicle tissue to characterize RUNX2 chromatin occupancy at the *FAM20A* and *FAM83H* locus.

Motif enrichment analysis using HOMER identified significant enrichment of the RUNX2-binding motif in the target regions (*p* = 1 × 10^−270^). The RUNX2 motif was detected in 20.87% of target sequences, compared with 16.78% of background sequences, suggesting an enrichment of RUNX2-associated DNA-binding sites in these regions (Figure 6A).

Visualization of the CUT&Tag profiles revealed prominent RUNX2 enrichment around the *FAM20A* TSS at chr17:68,601,367. This enrichment was consistently observed across three independent biological replicates (Figure 6B, Table 2). In contrast, analysis of the *FAM83H* locus did not reveal a comparable focal RUNX2 enrichment at the examined promoter/TSS-associated region. Although CUT&Tag signals were distributed across the *FAM83H* locus and surrounding genomic regions, no promoter-associated enrichment was consistently detected across the three biological replicates (Figure 6C). Thus, under these conditions, promoter/TSS-associated RUNX2 occupancy was evident at *FAM20A*, with no comparable focal occupancy at the examined *FAM83H* region. However, this does not exclude RUNX2 regulation of *FAM83H* through distal regulatory elements or indirect mechanisms.

### 2.7. RUNX2 Knockdown Is Accompanied by Reduced FAM20A Expression in Dental Follicle Cells

To further assess the regulatory relationship between RUNX2 and FAM20A, three distinct *RUNX2*-targeting siRNAs were examined in human dental follicle cells. All three siRNAs reduced *RUNX2* expression and were accompanied by consistent reductions in *FAM20A* expression, whereas *FAM83H* expression showed no comparable decrease (Figure 6D). The reduction in *FAM20A* expression was consistent across the three independent *RUNX2*-targeting siRNAs. Together, promoter/TSS-associated RUNX2 occupancy and reduced *FAM20A* expression following *RUNX2* knockdown support *FAM20A* as a candidate RUNX2-regulated target gene in dental follicle cells and provide a basis for the working model shown in Figure 6E.

## 3. Discussion

The present study expands the phenotypic and mechanistic understanding of the relationship between amelogenesis imperfecta and abnormal tooth eruption. By integrating three newly characterized families, transcriptomic profiling of dental follicle tissue, histological localization of FAM83H and FAM20A, and RUNX2-associated regulatory experiments, our findings suggest that enamel formation and tooth eruption may be biologically connected.

Pathogenic variants in *FAM83H* gene classically cause ADHCAI [10], characterized by relatively preserved enamel thickness, defective mineralization, and rapid post-eruptive enamel loss [29]. In contrast, biallelic variants in *FAM20A* cause ERS, which is typically associated with severe enamel hypoplasia or aplasia, pulp calcifications, gingival abnormalities, nephrocalcinosis, and delayed or failed tooth eruption [12,30,33]. In our study, *FAM83H*-associated ADHCAI showed predominantly enamel-centered abnormalities, with reduced enamel radiodensity and relatively preserved dentin which has been reported [10]. In contrast, *FAM20A*-associated disease exhibited more extensive enamel defects and tooth eruption failure, consistent with previous radiographic and histologic findings [8,33]. These genotype-specific CBCT features may aid radiographic differentiation between the two conditions.

The combined case analysis further showed distinct gene-specific eruption patterns, with abnormalities documented in all evaluable *FAM20A* cases, typically involving multiple teeth, but less frequently and more selectively in *FAM83H* cases [4,5]. 

To explore the biological basis underlying these gene-specific eruption phenotypes, previous studies of FAM20A and FAM83H provide important tissue and functional context. FAM20A is predominantly expressed in odontogenic epithelial compartments, including ameloblasts, and remains detectable in the reduced enamel epithelium during tooth eruption [6,34]. As a pseudokinase that activates FAM20C-dependent extracellular protein phosphorylation [35], FAM20A deficiency has been associated with dysregulation of Wnt-related signaling and impaired cellular functions, while epithelial-specific deletion of *Fam20a* disrupts Hertwig’s epithelial root sheath development, alters Wnt/β-catenin signaling and *Cdc42* expression, and delays osteoclast accumulation around erupting molars [36,37]. These findings suggest that epithelial FAM20A may participate in processes relevant to tooth eruption in addition to its established role in enamel formation.

Cytokeratins are expressed in odontogenic epithelial tissues, including the enamel organ, junctional epithelium, and epithelial rests of Malassez [38,39]. FAM83H regulates keratin cytoskeleton and desmosome organization, while AI-associated truncating variants in *FAM83H* disrupt these structures and alter FAM83H/CK1 localization [40,41,42].

Building on these previous findings, our tissue analyses further characterized the distribution of FAM20A and FAM83H in the dental follicle. IHC revealed prominent immunoreactivity for both proteins in epithelial components of the human dental follicle, with weaker staining in the surrounding connective tissue, and predominantly epithelial localization was also observed in developing mouse tooth germs. In parallel, exploratory bulk RNA-seq showed relatively low transcript levels of *FAM20A* and *FAM83H* in dental follicle tissue. These findings are not necessarily contradictory, as bulk RNA-seq reflects the transcriptomic profile of mixed cell populations and may therefore obscure cell type-specific expression, whereas IHC provides spatial information. Together, our findings extend the known epithelial distribution of FAM20A and FAM83H to the human dental follicle and underscore the importance of cellular heterogeneity when interpreting bulk transcriptomic data.

Several mechanisms have been proposed to explain tooth eruption, including root elongation, periodontal ligament traction, hydrostatic pressure, alveolar bone remodeling, and dental follicle-mediated regulation [43,44]. The dental follicle is predominantly ectomesenchymal and plays a central role in tooth development and eruption. It may also contain reduced enamel epithelium and persistent odontogenic epithelial remnants [17,18]. This cellular complexity is particularly relevant to tooth eruption, as the dental follicle coordinates coronal bone resorption and apical bone formation required for formation of the eruption pathway [17,45]. This process involves coordinated regulation of osteoclastogenesis and osteogenesis, including signaling through CSF-1 and the RANK/RANKL/OPG axis [45,46]. Enamel organ-derived epithelial structures are located in close proximity to the connective tissues in dental follicle and are thought to modulate their activity and osteoclastogenesis [47,48]. However, the functional contribution of these epithelial components and their interaction with the follicular mesenchyme during tooth eruption remain incompletely understood [17,38,39].

Within this complex cellular environment, RUNX2 is particularly relevant because it is a key transcriptional regulator of dental follicle biology and tooth eruption, coordinating osteogenic differentiation and osteoclast-inductive signaling [49,50]. The presence of FAM20A and FAM83H in epithelial components of the dental follicle therefore prompted us to examine whether a regulatory relationship exists between RUNX2 and either of these genes. CUT&Tag identified reproducible RUNX2 occupancy at the *FAM20A* promoter/TSS-associated region, whereas no comparable focal occupancy was detected at the examined *FAM83H* promoter/TSS-associated region. Consistently, *RUNX2* knockdown using three distinct siRNAs was accompanied by reduced FAM20A expression, whereas FAM83H showed no comparable decrease. Together, these findings support FAM20A as a candidate RUNX2-regulated target in dental follicle cells.

### 3.1. Limitations

The RNA-seq analysis was based on tissues from a single impacted third molar and lacked donor-level replication. The *RUNX2* knockdown experiment was performed using a single hDFC preparation, with three experimental replicates derived from separately maintained cell cultures and independently treated. Additional protein-level validation would further strengthen the findings. Functional studies related with *FAM20A* and *FAM83H* mutants should be performed. In addition, although CUT&Tag and RUNX2 knockdown support a regulatory relationship between RUNX2 and *FAM20A*, targeted chromatin assays and/or promoter-reporter experiments will be required to establish direct transcriptional regulation. Further functional studies using FAM20A and FAM83H mutant models are also needed to determine their roles in dental follicle function and tooth eruption. The molecular mechanisms underlying the role of FAM83H in tooth eruption remain to be elucidated.

### 3.2. Conclusions

Our findings suggest potential roles of FAM20A and FAM83H in tooth eruption beyond their established functions in enamel formation and identify FAM20A as a candidate RUNX2-regulated target in dental follicles. These observations support a working, testable model in which persistent epithelial components expressing FAM20A and FAM83H interact with the follicular mesenchyme and potentially influence local bone remodeling and tooth eruption. Within this model, FAM20A may be linked to the RUNX2-associated transcriptional network, whereas no similar relationship was evident for FAM83H. Further functional studies are needed to validate these proposed mechanisms.

## 4. Materials and Methods

### 4.1. Participants and Clinical Examinations

All participants underwent evaluation at the Clinic of Oral Rare Diseases and Genetic Diseases, School of Stomatology, the Fourth Military Medical University. Written informed consent was obtained from adult participants and from the legal guardians of minor participants. All investigations were approved by the Institutional Review Board (approval number: IRB-REV-2022182; 1 December 2022).

Medical and dental histories were obtained, followed by extraoral and intraoral examination. Panoramic radiography, Cone Beam Computed Tomography (CBCT), or computed tomography (CT) were applied for tooth or renal changes.

### 4.2. Gene Analysis

Genomic DNA was extracted from peripheral blood. Whole-exome sequencing (WES) or whole-genome sequencing (WGS) was performed by BGI (Shenzhen, China) on the BGISEQ-500 or DNBSEQ platform with a 150-bp paired-end strategy. The identified candidate variants were screened bioinformatically, followed by PCR and Sanger sequencing for confirmation and segregation analysis as previously reported [3]. *FAM83H* variants were interpreted using transcript NM_198488.5. In the *FAM20A* family, variants were described using NM_017565.4; direct sequencing identified c.755_757delTCT (p.Phe252del) and c.826C>T (p.Arg276Ter). TA cloning of PCR products was used to demonstrate the two variants on different alleles [3].

### 4.3. Updated Retrospective Literature Analysis

An updated retrospective literature review was performed to identify human *FAM20A*- and *FAM83H*-associated AI cases reported after the period covered by our previous eruption studies [4,5]. PubMed was searched from 1 January 2023 to 6 September 2026 using the following terms: (FAM20A OR FAM83H) AND (amelogenesis imperfecta OR enamel renal syndrome OR tooth eruption OR delayed eruption OR failure of eruption OR unerupted tooth OR impacted tooth). Inclusion and exclusion criteria were as previously reported [4,5]. Briefly, studies [27,28,29,30,31,32] were included, all of which (1) reported individuals with molecularly confirmed variants in *FAM20A* or *FAM83H*; (2) described a recognizable enamel phenotype; and (3) provided sufficient clinical or radiographic information to evaluate eruption status. Reviews, animal or in vitro studies, duplicate reports of the same cases, reports without genetic confirmation or evaluable eruption information were excluded.

For each eligible individual, we extracted the gene, variant, enamel and eruption phenotypes, and associated dental or systemic findings. Eruption status, including delayed or failed eruption, was determined from the original clinical descriptions and available radiographs. Delayed permanent eruption was defined as a patient’s age exceeding the corresponding tooth-specific mean eruption age in Chinese normative data by >2 SD [4,5]. Only individuals with sufficient information to determine eruption status, considering age or dentition stage, were included in the denominator; those with at least one eruption abnormality constituted the numerator. Missing data were not imputed [4,5].

The historical datasets [4,5], newly identified cases from the literature, and the three AI families in the present study were combined to calculate updated gene-specific proportions. The three newly characterized families were included as phenotypic exemplars complementing the larger retrospective dataset and were distinguished from previously reported cases in the analysis.

### 4.4. Human Dental Follicle Collection and Related Experimental Analyses

Dental follicle tissues were obtained from eight healthy donors aged 18–22 years undergoing surgical extraction of impacted third molars. The following analyses were performed on the tissues. Specimens from independent donors were allocated to different experiments: one specimen was used for bulk RNA-seq (*n* = 1), three for histological and immunohistochemical analyses (*n* = 3), three for CUT&Tag analysis (*n* = 3), and one for primary human dental follicle cell (hDFC) culture (*n* = 1).

#### 4.4.1. RNA Extraction and Transcriptome Sequencing

Dental follicle, dental pulp, and apical papilla were obtained from a single extracted impacted third molar. The total RNA were extracted and processed bulk transcriptome sequencing was performed on the DNBSEQ platform (BGI, Shenzhen, China). Transcript abundance is summarized as fragments per kilobase of transcript per million mapped reads (FPKM). The three tissues were compared descriptively for epithelial-associated transcripts and visualized by heat-map analysis. Given the single-tooth sampling, the RNA-seq data were analyzed as an exploratory, descriptive tissue-level comparison.

#### 4.4.2. Immunohistochemistry

Human dental follicles surrounding impact third molars were collected (*n* = 3) and fixed in 4% paraformaldehyde (Biosharp, Beijing, China) in a 0.01 mol/L phosphate buffer (pH 7.4). After fixation, the specimens were dehydrated and embedded in paraffin. Then, 4μm sections were cut, and immunohistochemistry staining was performed using a rabbit two-step enhanced polymer detection system (ZSGB-BIO, Beijing, China), followed by DAB staining (ZSGB-BIO, Beijing, China) and hematoxylin counterstaining (Leagene Biotechnology, Beijing, China). Primary antibodies included anti-FAM20A (1:200; Cat. No. 25258-1-AP; Proteintech, Rosemont, IL, USA) and anti-FAM83H (1:100; Cat. No. DF15132; Affinity Biosciences, Cincinnati, OH, USA) antibodies. Antibody specificity was supported by the manufacturers’ validation data and previously reported applications. Negative controls were processed in parallel with omission of the primary antibody. Staining was assessed qualitatively based on the distribution and relative intensity of immunoreactivity in epithelial and connective tissue components; no quantitative scoring was performed.

In addition, histopathologically confirmed dentigerous cyst specimens from three independent patients (*n* = 3) were obtained from archival clinical pathology samples. Formalin-fixed, paraffin-embedded tissue blocks were used for histological and immunohistochemical analyses, with all patient information de-identified before analysis.

#### 4.4.3. RUNX2 CUT&Tag Sequencing and Peak Annotation

RUNX2 chromatin occupancy in dental follicle tissue was profiled by Cleavage Under Targets and Tagmentation (CUT&Tag) [51] using a commercial service (Novogene, Beijing, China), with three biological replicates (*n* = 3). Briefly, cells were immobilized on concanavalin A-coated beads, permeabilized with digitonin, and sequentially incubated with an anti-RUNX2 primary antibody (Cat. No. ab236639; Abcam, Cambridge, UK) and pA-Tn5 transposase. Tagmented DNA was PCR-amplified, purified using AMPure beads, and assessed using an Agilent 2100 Bioanalyzer (Agilent Technologies, Santa Clara, CA, USA). Libraries were sequenced on an Illumina NovaSeq platform (Illumina, Inc., San Diego, CA, USA) to generate 150-bp paired-end reads.

Raw reads were quality-filtered with fastp and aligned to the human reference genome (GRCh38/hg38) using BWA. For locus-level analyses, FAM20A and FAM83H were annotated using the reference transcripts NM_017565.4 and NM_198488.5, respectively, with genomic coordinates based on the GRCh38/hg38 genome assembly. Peaks were called using MACS2 with a significance threshold of q < 0.05. Signal tracks were normalized to reads per million mapped reads (RPM) for comparison and genome-browser visualization. Reproducibility was assessed based on concordant peak enrichment at overlapping or closely adjacent genomic regions across all three biological replicates. Peaks were annotated using ChIPseeker, and motif and functional enrichment analyses were performed using HOMER, GOseq, and KOBAS.

### 4.5. Primary Dental Follicle Cell Culture and Related Experiments

#### 4.5.1. Primary Culture

Primary hDFCs were isolated from one surgically extracted impacted third molar (*n* = 1), washed with phosphate-buffered saline (PBS; Biosharp, Beijing, China), and minced into approximately 1 × 1 mm fragments under sterile conditions. The minced tissues were digested with 3 mg/mL collagenase type I and 4 mg/mL Dispase II (Thermo Fisher Scientific, Waltham, MA, USA) at 37 °C for 2 h. Then the cells were cultured in complete culture medium containing α-minimum essential medium (α-MEM; Gibco, Thermo Fisher Scientific, Waltham, MA, USA), 10% fetal bovine serum (FBS; VivaCell, Shanghai XP Biomed Ltd., Shanghai, China), and 1% Antibiotic-Antimycotic (100×; Gibco, Thermo Fisher Scientific, Waltham, MA, USA) at 37 °C in a humidified atmosphere containing 5% CO_2_. The culture medium was replaced 24 h after initial seeding and subsequently every 3 days. Adherent cells were expanded and passaged after reaching appropriate confluence and were used for subsequent experiments.

#### 4.5.2. siRNA Experiment and Transcriptome Sequencing

Small interfering RNA (siRNA) duplexes targeting human *RUNX2* were designed and synthesized by RIBOBIO (Guangzhou, China). Three distinct *RUNX2*-targeting siRNAs were used, with the following target sequences (5′–3′): #1, GCACTCCATATCTCTACTA; #2, GTTCAACGATCTGAGATTT; and #3, GTGGTCCTATGACCAGTCT. A non-targeting siRNA supplied by the same manufacturer was used as the negative control (NC). Cells were divided into two groups and transfected with either a non-targeting negative-control siRNA (NC) or RUNX2-specific siRNA using Lipofectamine™ 2000 Transfection Reagent (Invitrogen, Thermo Fisher Scientific, Carlsbad, CA, USA; Cat. No. 11668019) according to the manufacturer’s instructions. The negative control and each *RUNX2*-targeting siRNA group included three experimental replicates. For each group, the three experimental wells were established from separately maintained cell cultures originating from the same hDFC preparation and were treated independently.

After 48 h, RNA sequencing was performed as described above. Raw read counts were normalized across samples using the median-of-ratios size-factor method. Normalized counts for *RUNX2*, *FAM20A*, and *FAM83H* were extracted for selected-gene analysis and log2-transformed [log2(count + 1)]; this analysis was distinct from genome-wide differential-expression analysis. Expression values are summarized as mean ± SD, and expression ratios and percentage changes relative to NC were calculated. Exploratory comparisons among the technical replicate wells were performed using one-way ANOVA followed by Dunnett’s multiple-comparisons test, with each *RUNX2*-siRNA group compared with the NC group. Statistical analyses were performed using GraphPad Prism (version 11).

### 4.6. Other Experiments

C57BL/6 mice were obtained from the Animal Center of the Fourth Military Medical University in accordance with institutional guidelines and the ARRIVE 2.0 guidelines. Tooth germs and surrounding tissues were collected at postnatal days 0 (P0) (*n* = 3) and 7 (P7) (*n* = 3), fixed and decalcified as described above, and processed for IHC staining with primary antibodies against *FAM20A* or *FAM83H* as described above.

## Figures and Tables

**Figure 1:**
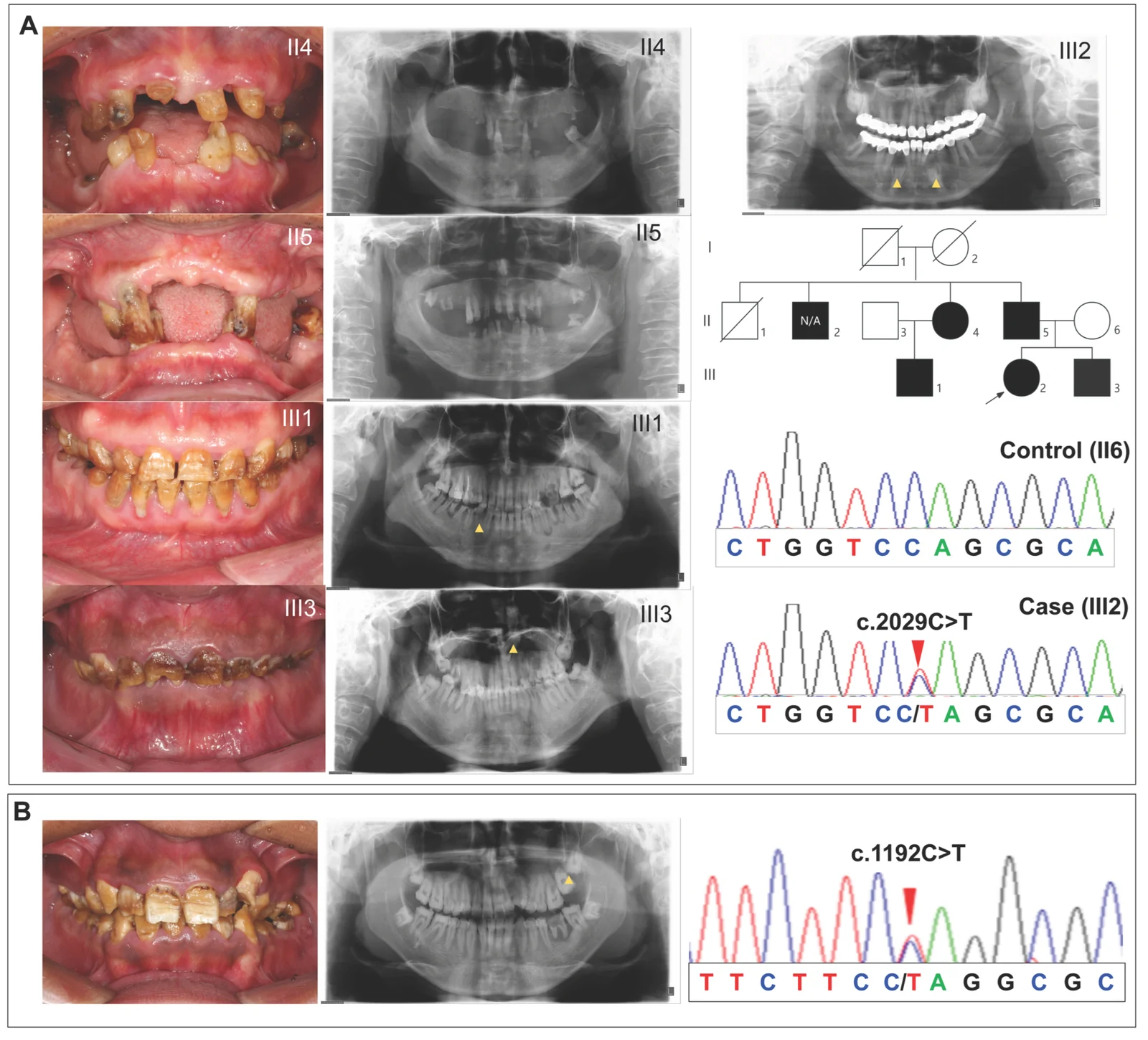
Clinical, radiographic, pedigree, and genetic findings in two families with FAM83H-associated amelogenesis imperfecta. (**A**) Family 1. Representative intraoral photographs and panoramic radiographs of affected family members (II4, II5, III1, III2, and III3) show generalized yellow to yellow-brown enamel discoloration, rough hypomineralized enamel, and reduced enamel radiodensity. Panoramic radiographs demonstrate unerupted canines in the proband III2 (teeth 33 and 43), III1 (tooth 43), and III3 (tooth 23), as indicated by yellow arrowheads. The pedigree is consistent with autosomal dominant inheritance. Sanger sequencing confirmed the heterozygous variant in *FAM83H* NM_198488.5: c.2029C>T [p.(Gln677Ter)] (red arrowhead) in affected individuals, whereas tested unaffected relatives were variant-negative; representative chromatograms from the proband (III2) and an unaffected control (II6) are shown. (**B**) Family 2. The proband exhibited generalized rough yellow-brown and groove-like enamel defects. Panoramic radiography showed abnormal eruption of tooth 27 (yellow arrowhead). Sanger sequencing identified the heterozygous variant in *FAM83H* NM_198488.5: c.1192C>T [p.(Gln398Ter)] in the proband (red arrowhead). Filled symbols indicate affected individuals, open symbols indicate unaffected individuals, the black arrow denotes the proband, and N/A indicates that genetic testing was unavailable.

**Figure 2:**
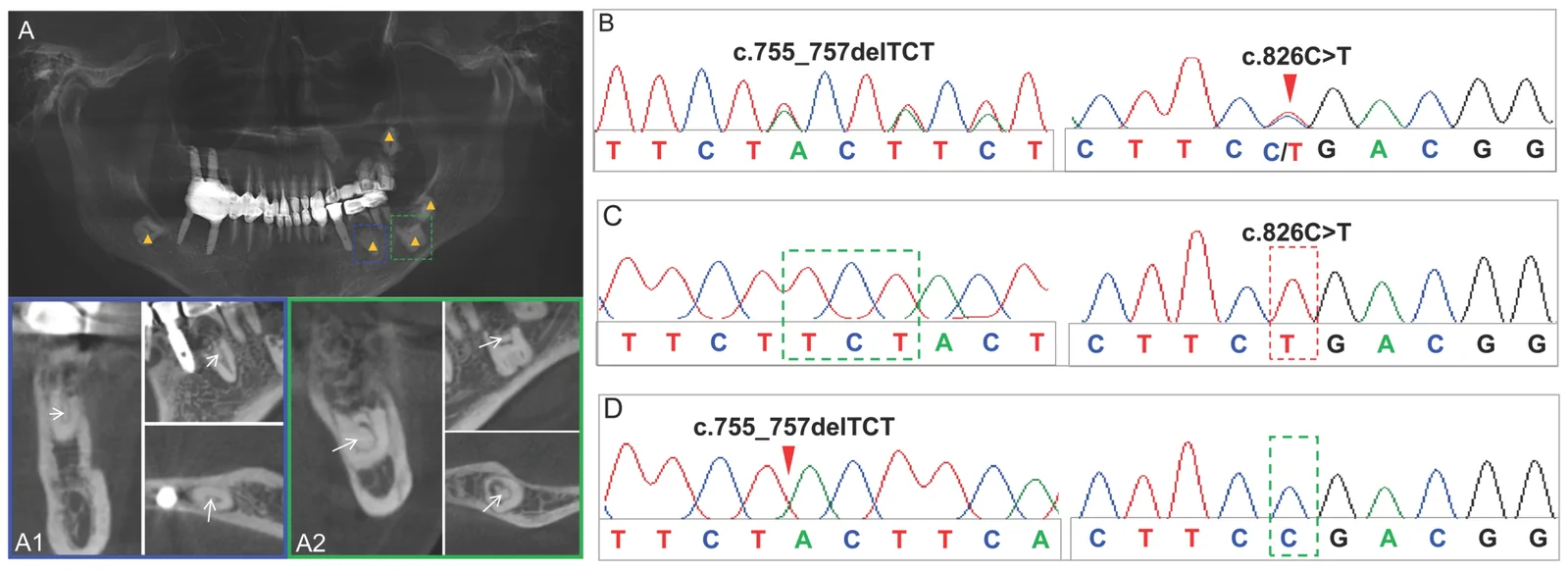
Clinical, radiographic, and molecular findings in the *FAM20A* proband with ERS. (**A**) Panoramic radiograph showing full-mouth prosthodontic rehabilitation and multiple unerupted teeth (25, 28, 35, 37, 38, and 48; yellow arrowheads) in the proband. (**A1**,**A2**) Representative CBCT images of unerupted teeth 35 (blue outline) and 37 (green outline) demonstrate pulp calcifications (white arrows) and abnormal crown. (**B**) Sanger sequencing confirmed compound heterozygous variants in *FAM20A* NM_017565.4: c.755_757delTCT [p.(Phe252del)] and c.826C>T [p.(Arg276Ter)] in the proband. The red arrowhead indicates the heterozygous c.826C>T substitution. (**C**,**D**) Cloning-based allelic separation followed by Sanger sequencing demonstrated that the two variants were located on different alleles (in trans). (**C**) One allele carried c.826C>T (highlighted by red dashed boxe) while retaining the wild-type sequence at c.755_757 (highlighted by green dashed boxe). (**D**) The other allele carried c.755_757delTCT while retaining the wild-type C nucleotide at c.826. Red arrowheads and green dashed boxes indicate the respective variant and wild-type sequence positions.

**Figure 3:**
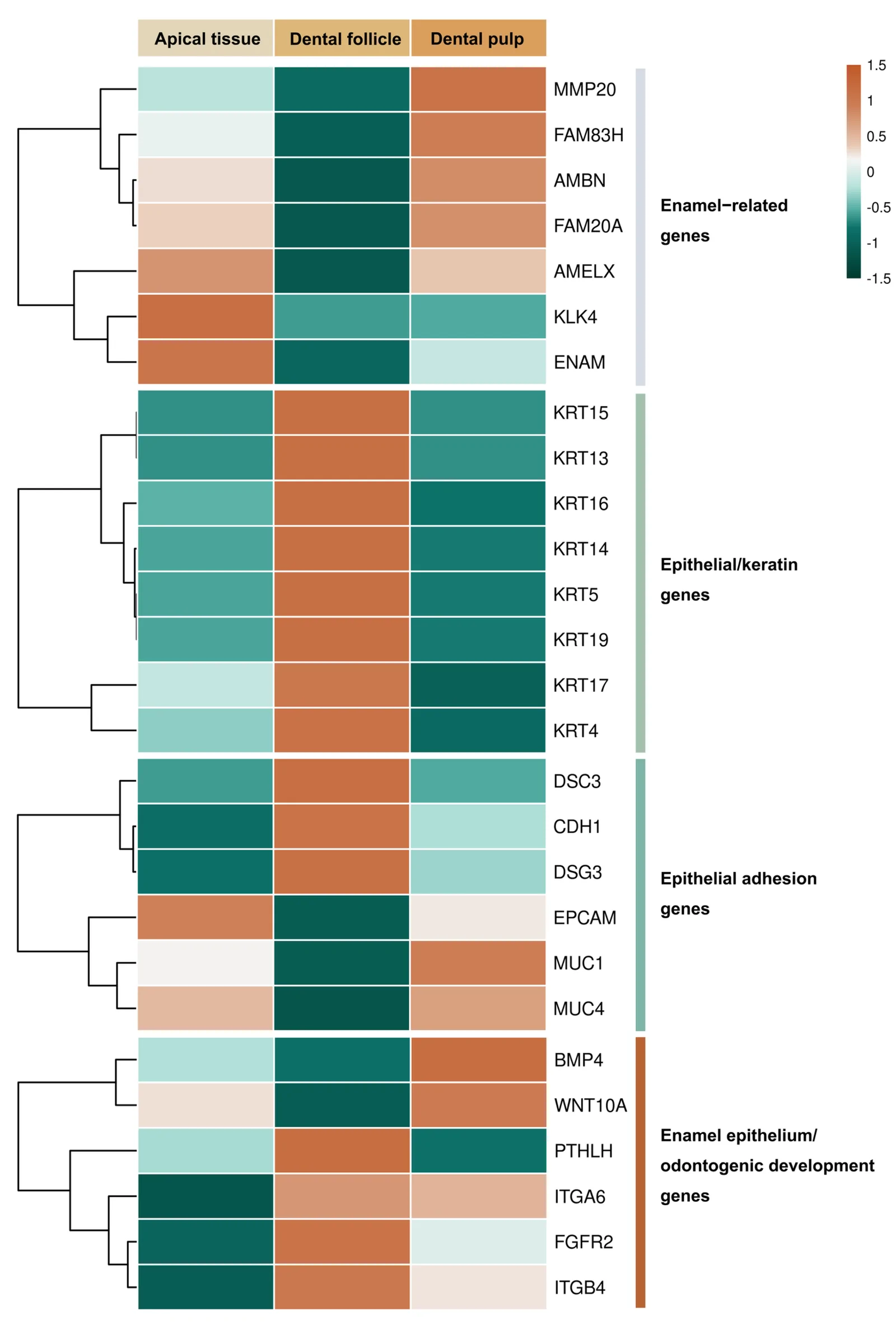
Heatmap of *FAM20A*, *FAM83H*, and Genes Related to Enamel Formation and Epithelial Functions. RNA-sequencing data are presented as heatmap profiles of selected genes in apical papilla, dental follicle, and dental pulp tissues (*n* = 1). *FAM20A* and *FAM83H* were analyzed together with genes associated with enamel formation (*MMP20, AMBN*, *AMELX*, *KLK4*, and *ENAM*), epithelial/keratin identity (*KRT15*, *KRT13*, *KRT16*, *KRT14*, *KRT5*, *KRT19*, *KRT17*, and *KRT4*), epithelial adhesion (*DSC3*, *CDH1*, *DSG3*, *EPCAM*, *MUC1*, and *MUC4*), and enamel epithelium/odontogenic development (*BMP4*, *WNT10A*, *PTHLH*, *ITGA6*, *FGFR2*, and *ITGB4*). Genes were hierarchically clustered within the indicated functional groups. The color scale represents standardized relative expression levels, with orange indicating higher expression and green indicating lower expression.

**Figure 4:**
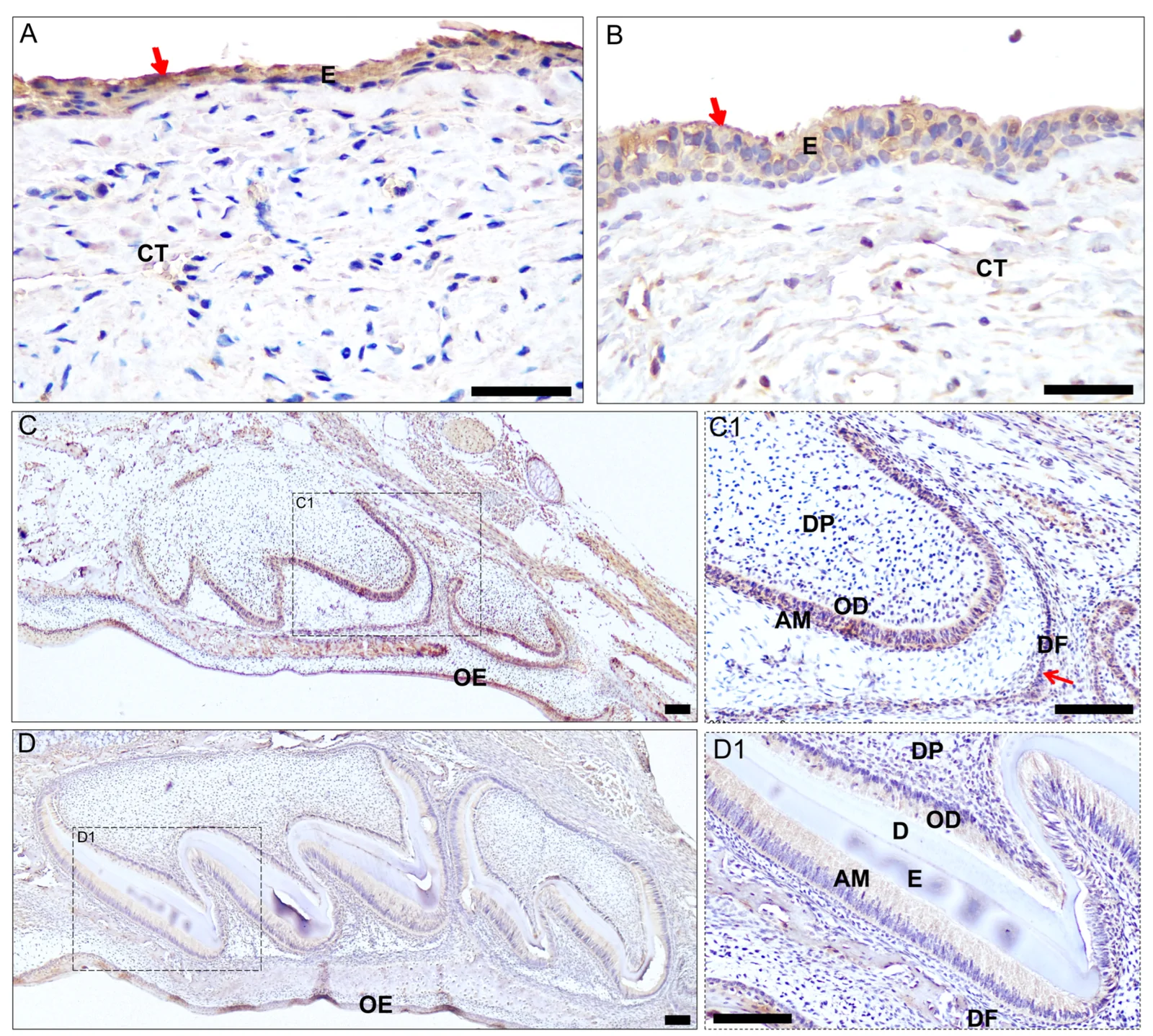
Immunohistochemical localization of FAM83H in human dental follicles and developing mouse tooth germs. (**A**) Dental follicle (DF) surrounding an impacted human third molar showing strong FAM83H immunoreactivity in odontogenic epithelial remnants (E; red arrow) and weak staining in the surrounding follicular connective tissue (CT). (**B**) Dentigerous cyst from the maxillary canine region showing strong FAM83H immunoreactivity in the pathological cystic epithelial lining (E; red arrow) and weak staining in the underlying CT. (**C**,**C1**) Mouse tooth germ at postnatal day 0 (P0), showing prominent FAM83H immunoreactivity in epithelial components of the enamel organ, including ameloblasts, as well as odontoblasts, with weak staining in DF. (**C1**) shows a higher-magnification view of the boxed region in (**C**). (**D**,**D1**) Mouse tooth germ at postnatal day 7 (P7), showing FAM83H immunoreactivity similar predominantly in odontogenic epithelial/enamel-forming compartments, with no prominent staining in DF. (**D1**) shows a higher-magnification view of the boxed region in (**D**). E, epithelium; CT, connective tissue; OE, oral epithelium; DP, dental papilla; AM, ameloblasts; OD, odontoblasts; DF, dental follicle. Scale bars: 50 μm (**A**,**B**); 100 μm (**C**,**C1**,**D**,**D1**). *n* = 3.

**Figure 5:**
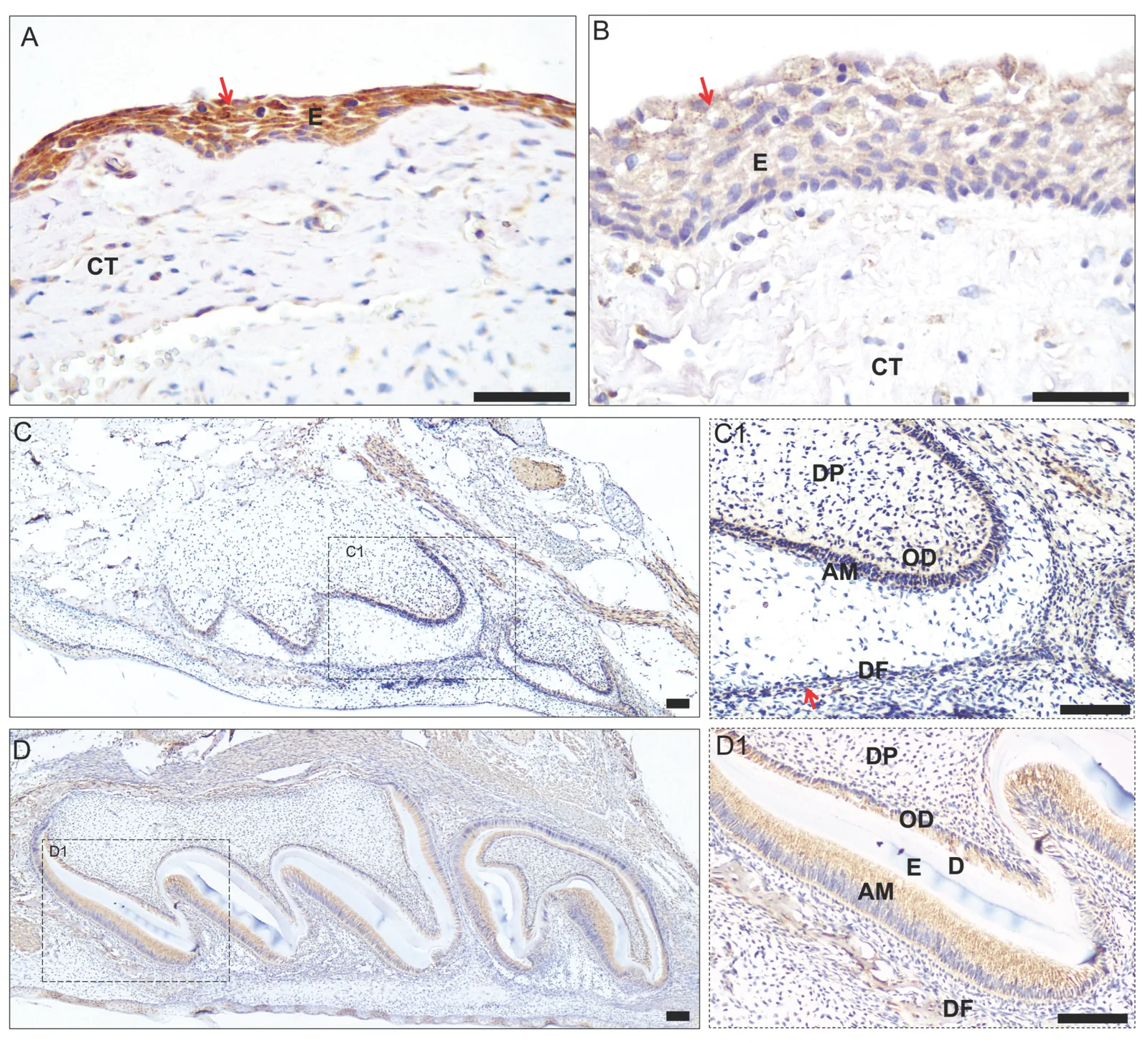
Immunohistochemical localization of FAM20A in human dental follicles and developing mouse tooth germs. (**A**,**B**) FAM20A immunohistochemistry in the dental follicle (DF) surrounding an impacted third molar (**A**) and in a dentigerous cyst (**B**). Strong FAM20A immunoreactivity is observed in the epithelial components of the DF (**A**) and the pathological cystic epithelial lining (E, red arrows) (**B**), with weak staining in the adjacent connective tissue (CT). (**C**,**D**) FAM20A expression in mouse tooth germs at P0 (**C**) and P7 (**D**). Strong immunoreactivity is observed in the oral/odontogenic epithelium and enamel-organ/ameloblast-lineage cells, with weaker staining in mesenchymal and mineralizing compartments. (**C1**,**D1**) Higher-magnification views of the boxed regions in ((**C**) and (**D**)), respectively, showing FAM20A expression in ameloblasts (AM), odontoblasts (OD), dental papilla (DP), and DF. E, epithelium in (**A**,**B**) and enamel in (**D1**); CT, connective tissue; AM, ameloblasts; OD, odontoblasts; DP, dental papilla; DF, dental follicle; D, dentin. Scale bars: 50 μm (**A**,**B**); 100 μm (**C**,**C1**,**D**,**D1**). *n* = 3.

**Figure 6:**
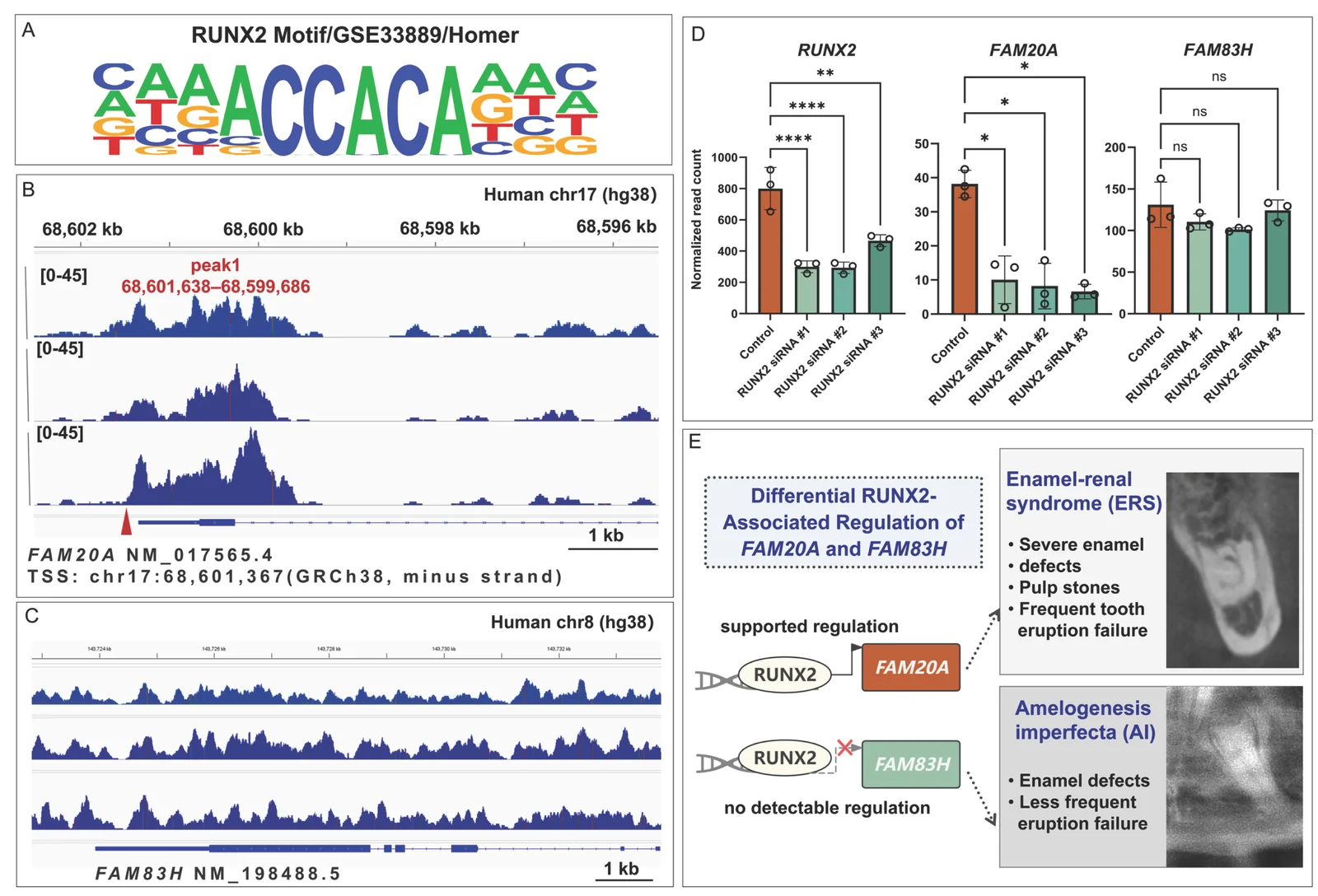
Evidence supporting *FAM20A* as a candidate RUNX2-regulated target gene in dental follicle cells. (**A**) Enrichment of the RUNX2 DNA-binding motif in target genomic regions. The sequence logo represents the RUNX2-binding motif identified by HOMER motif enrichment analysis. Nucleotide heights indicate the information content at each position. The RUNX2 motif was detected in 20.87% of target sequences and 16.78% of background sequences (*p* = 1 × 10^−270^). (**B**) RUNX2 CUT&Tag profiles at the human *FAM20A* locus (NM_017565.4; chr17, GRCh38) from three independent biological replicates. A prominent RUNX2-enriched region (peak 1, chr17:68,599,686–68,601,638) encompasses the *FAM20A* transcription start site (TSS; chr17:68,601,367, red arrowhead), indicating reproducible RUNX2 occupancy at the TSS-associated region. *n* = 3. (**C**) RUNX2 CUT&Tag profiles at the human *FAM83H* locus (NM_198488.5; chr8, GRCh38) from the same three biological replicates. In contrast to *FAM20A*, no prominent and reproducible focal RUNX2 enrichment was observed at the *FAM83H* promoter/TSS-associated region. *n* = 3. (**D**) Effects of three independent *RUNX2* siRNAs on *RUNX2*, *FAM20A*, and *FAM83H* expression. Normalized RNA-seq read counts are shown for three replicates per group. Statistical analysis was performed on log2-transformed normalized counts using one-way ANOVA followed by Dunnett’s test versus NC. * *p* < 0.05, ** *p* < 0.01, **** *p* < 0.0001; ns, not significant. The three wells were prepared from separate cell cultures rather than a single batch and were independently treated. (**E**) Proposed working model in which RUNX2-associated regulation of *FAM20A* may provide a potential molecular link between enamel defects and tooth eruption failure. No comparable regulatory relationship was evident for *FAM83H* under the experimental conditions. ERS ascertainment partly depends on eruption failure.

**Table 1:** Comparison of tooth-eruption abnormalities associated with *FAM20A* and *FAM83H.*

Characteristic	FAM20A	FAM83H
Historical cohort with eruption abnormalities, *n*/N (%)	20/20 (100%) [5]	12/50 (24.0%) [4]
Unerupted teeth in historical cohort, *n* (mean/patient)	209 (10.5) [5]	34 (2.83) [4]
2023–2026 update cases, *n*/N (%)	15/16(93.8%)	2/2 (100%)
Present case(s), *n*/N (%)	1/1 (100%)	4/6 (66.7%)
Overall eruption abnormalities, *n*/N (%)	36/37 (97.3%)	18/58 (31.0%)
Distribution of affected teeth	Multiple tooth types affected; second molars and mandibular canines affected in >50%	Selective involvement of canines and second molars

Note: Data were integrated from previously published datasets [4,5], eligible reports published during 2023–2026, and the present genetically confirmed cases. n: the number of patients with eruption abnormalities with clear genetic background; N: total patients with clear eruption status and pathogenic variants in *FAM20A* or *FAM83H*.

**Table 2:** RUNX2 CUT&Tag peaks annotated to FAM20A promoter regions in dental follicle.

Peak Region	Peak Length (bp)	Annotation	Transcript ID	TSS Position	Distance to TSS (bp)	Count, Mean ± SD (*n* = 3)
chr17:68599686–68601638	1952	Promoter (≤1 kb)	ENST00000592554	68601367	0	208.0 ± 44.4
chr17:68613826–68614123	297	Intron	ENST00000592554	68601367	−12,459	21.0 ± 7.8
chr17:68537423–68537638	215	Promoter (2–3 kb)	ENST00000619787	68540577	2939	20.7 ± 3.8
chr17:68539440–68540257	817	Promoter (≤1 kb)	ENST00000619787	68540577	320	45.7 ± 15.1
chr17:68540758–68541362	604	Promoter (≤1 kb)	ENST00000619787	68540577	−181	57.0 ± 13.0
chr17:68542667–68542937	270	Promoter (≤1 kb)	ENST00000375556	68541991	−676	22.3 ± 3.2
chr17:68548165–68548379	214	Intron	ENST00000590873	68543669	−4496	18.0 ± 4.4
chr17:68561906–68562120	214	Intron	ENST00000226094	68558390	−3516	16.7 ± 3.1
chr17:68563012–68563335	323	Intron	ENST00000226094	68558390	−4622	13.3 ± 6.1
chr17:68564625–68564969	344	Intron	ENST00000226094	68558390	−6235	28.0 ± 9.2
chr17:68569752–68570287	535	Intron	ENST00000226094	68558390	−11,362	16.3 ± 10.5

## Data Availability

The data supporting the findings of this study are available from the corresponding author upon reasonable request, subject to applicable ethical and privacy restrictions.
